# Global Transcriptomic Profiling of Pulmonary Gene Expression in an Experimental Murine Model of *Rickettsia conorii* Infection

**DOI:** 10.3390/genes10030204

**Published:** 2019-03-08

**Authors:** Hema P. Narra, Abha Sahni, Kamil Khanipov, Yuriy Fofanov, Sanjeev K. Sahni

**Affiliations:** 1 Department of Pathology, University of Texas Medical Branch, Galveston, TX 77555, USA; absahni@utmb.edu; 2 Department of Pharmacology, University of Texas Medical Branch, Galveston, TX 77555, USA; kakhanip@utmb.edu (K.K.); yufofano@utmb.edu (Y.F.)

**Keywords:** *Rickettsia*, C3H/HeN mice, RNA sequencing, transcriptome, ingenuity pathway analysis, antimicrobial peptides, guanylate-binding proteins, calgranulins, ubiquitination

## Abstract

Mediterranean spotted fever develops from an infection with *Rickettsia conorii*, an obligate intracellular, Gram-negative, endotheliotropic, and tick-transmitted bacterial pathogen, and is an acute, febrile illness that can progress to life-threatening complications if not diagnosed and treated early with effective antibiotics. Despite significant morbidity and mortality, little is known about changes in gene expression that determine the host responses during in vivo infection. We have investigated the transcriptional landscape of host lungs as a prominently affected organ system in an established murine model of infection by RNA-sequencing. Ingenuity pathway analysis resulted in the identification of 1332 differentially expressed genes and 292 upstream regulators. Notably, genes encoding for ubiquitin D, aconitate decarboxylase, antimicrobial peptides, calgranulins, cytokines and chemokines, and guanylate binding proteins were highly up-regulated, whereas those involved in hemoglobin biosynthesis and heme homeostasis were significantly down-regulated. Amongst response regulators, nucleotide-binding oligomerization domain-containing protein 2 and killer cell lectin-like receptors were differentially expressed, and gene clustering revealed eukaryotic initiation factor-2, oxidative phosphorylation, and ubiquitination as the predominantly activated biological pathways. Collectively, this first global transcriptomic profiling has identified *R. conorii*-induced regulation of novel genes and pathways in the host lungs, further in-depth investigation of which will strengthen our understanding of the pathogenesis of human rickettsioses.

## 1. Introduction

Rickettsioses caused by obligate intracellular pathogens in the family *Rickettsiaceae* are emerging and reemerging infectious diseases with considerable historical significance, and they are amongst the oldest known arthropod-borne zoonotic infections [1,2]. Spotted fever group rickettsioses include Rocky Mountain spotted fever (RMSF) caused by *Rickettsia rickettsii* in the United States and Latin Americas, and Mediterranean spotted fever (MSF) or Boutoneusse fever caused by *R. conorii* in Europe, Africa, and Asia. They continue to threaten public health in different parts of the world, and vaccines are currently unavailable for these diseases [1]. Over the past decade, increased incidence of rickettsioses across the globe has been reported (with the exception of Antarctica), and warmer temperatures attributed to global environmental changes have been predicted to result in increased widespread distribution of rickettsial diseases afflicting humans [3]. It is important to note that epidemiological data on human rickettsioses from many regions of the world remains ambiguous. Serological surveys are unable to differentiate prior exposures that lead to an antibody response from recent infection. Moreover, a number of infected cases only progress to mild, self-limiting illness and, thus, the infected cases do not seek healthcare.

Mediterranean spotted fever is an acute, febrile illness with non-specific initial flu-like symptoms. This can progress to life-threatening complications as a result of aberrations in vascular permeability leading to fluid accumulation in vital organs, thrombocytopenia, and vascular inflammation and dysfunction that manifests as rickettsial vasculitis. Multi-organ failure, with considerable morbidity and mortality, can result if not diagnosed accurately and treated early with effective antibiotics [1,4]. Upon successful transmission to a mammalian host through a tick bite, *R. conorii* primarily targets the endothelial cell lining of the vasculature, and lungs are one of the major organ systems adversely affected during in vivo infection [5]. Several in vitro studies with cultured human endothelial cells have implicated the involvement of numerous molecular mediators in dictating host response mechanisms, such as infection-induced signaling, activation of innate immunity, production of pro-inflammatory cytokines, intracellular rickettsial killing, oxidative stress, and antioxidant defense [6,7,8,9,10,11]. However, in vivo pathophysiology of rickettsial infections is not completely understood, owing to complex interactions involving other host cell types and cellular cross-talk as the ultimate determinant of host responses, severity of disease, and outcome of infections. To this end, animal models of rickettsial diseases offer a valuable resource to understand the pathologic basis of disease, investigate immune mechanisms involved in pathogen clearance from the host, decipher the roles of vector-associated factors in disease transmission and progression, and to test the efficacy of potential vaccines [12,13]. Several murine models based on the degree of susceptibility of different host strains, routes of infection, and manifestation of overt signs of disease have been developed for different pathogenic species to better understand the pathophysiology of rickettsial infections [14,15,16].

Currently, infection of susceptible C3H/HeN mice with *R. conorii* is one of the best available animal models to mimic the salient manifestations of human rickettsial infections, as exemplified by its ability to recapitulate the expression of tumor necrosis factor-α (TNF-α), interferon-γ (INF-γ), chemokine (C-C motif) ligand 5 (*ccl5*/*RANTES*), indoleamine-2,3-dioxygenase (*Ido*), and inducible nitric oxide synthase (iNOS) as observed in human patients with MSF [17,18]. Using this well-established model, other studies have further elucidated the roles of host receptors such as fibroblast growth factor receptor 1 (FGFR1) and toll-like receptors TLR-2 and TLR-4 in rickettsial invasion, as well as associated signaling molecules such as myeloid differentiation factor MyD88 in host responses and pathogenesis [19,20,21,22]. Additionally, the roles for natural killer (NK) cells and CD4+ and CD8+ T-cells in protective immunity and rickettsial clearance from the host have also been investigated [23,24]. Despite this noteworthy progress, correlation between host responses, clinical signs/symptoms, and treatment outcomes remains limited because there is a lack of complete understanding of the changes in host transcriptome and immune modulation during rickettsial infection.

Deep sequencing-based approaches, such as RNA sequencing (RNA-seq), offer a powerful tool for unraveling transcriptome complexity and deducing differentially regulated canonical pathways during host–pathogen interplay. It is increasingly and effectively exploited for transcriptional profiling of different organs and tissues during bacterial, fungal, and viral infections [25,26,27]. In the present study, we have applied an RNA-seq approach to decode the global transcriptional landscape of lung tissue during in vivo *R. conorii* infection in susceptible experimental hosts to identify the receptors, regulators, and pathways that are either activated or compromised during rickettsial infection. As expected, our findings reveal a distinct transcriptional profile in mouse lungs during *R. conorii* infection. Using ingenuity pathway analysis (IPA), we have further identified 1332 genes and 292 upstream regulators exhibiting altered gene expression, suggesting the involvement of a complex regulatory network punctuated by the potential for significant mechanistic cross-talk during host–pathogen interactions in vivo.

## 2. Material and Methods

### 2.1. Mammalian Cell Culture and Preparation of Rickettsia conorii Stocks

Vero E6 cells derived from the kidney of African green monkeys and purchased from American type culture collection (ATCC^®^CRL-1586^TM^) were grown to ~90% confluence in Dulbecco’s Modified Eagle Medium supplemented with heat-inactivated fetal bovine serum (5% *v*/*v*; Aleken Biologicals, Nash, TX, USA) and L-glutamine (10 mM; Thermo Fisher Scientific, Waltham, MA, USA). The monolayers of cultured Vero cells were infected with *R. conorii* (Malish7 strain) and were incubated at 35 °C in an atmosphere of 95% O_2_ and 5% CO_2_ until approximately 80% of the monolayer was heavily infected (>50 rickettsiae/cell). *R. conorii* was released by gently lysing infected Vero cells with sterile 4 mm glass beads (Thermo Fisher Scientific) and purified by differential centrifugation. The pathogen stocks thus prepared were stored as aliquots of ≤0.5 mL at −80 °C until further use [28]. The infectivity titer of purified *R. conorii* stocks was determined by citrate synthase (*gltA*)-based quantitative PCR followed by plaque formation assay on Vero cells [29].

### 2.2. Mouse Model of Infection

All protocols used for the housing and infection of mice with *R. conorii*, post-infection monitoring, and collection of lungs from the mice were reviewed and approved by the Institutional Animal Care and Use Committee at the University of Texas Medical Branch (UTMB), Galveston, TX, USA. The University has a file with the Office of Laboratory Animal Welfare an approved Assurance Statement (#A3314-01). Six to eight-week old C3H/HeN male mice were purchased from The Jackson laboratory and acclimatized at our vivarium facility for a minimum of three days, during which they were assigned into experimental groups and baseline body weights were recorded. A group of animals was intravenously injected through the tail vein with a lethal dose of *R. conorii* (2.25 × 10^5^ pfu/mouse), while the control (mock-infected) group of mice were injected with an equal volume of sterile saline. Both mock- and *R. conorii*-infected mice were housed in designated cages inside the animal biosafety level 3 (ABSL3) suite and monitored on a daily basis for weight loss and external signs of disease. On day three post-infection (p.i.), mice were euthanized, and lungs were collected aseptically and stored immediately in an RNAlater^TM^ solution at −20 °C for extraction of total DNA and RNA. Three days p.i. represents the earliest time at which mice display clinical signs of disease after the incubation period. There is significant pulmonary infection based on the detection of a number of *R. conorii* in the lungs of infected mice as determined by SYBR^®^ Green-based quantitative PCR using rickettsial outer membrane protein (*ompA*) gene-specific primers and recovery of viable rickettsiae from the lungs [6,29].

### 2.3. Purification of Total RNA, Complementary DNA Library Preparation, and RNA Sequencing

Total RNA from a piece of mock- and *R. conorii*-infected mouse lungs was extracted using an established Tri-reagent protocol. Total RNA was treated with DNaseI (New England Biolabs, Ipswich, MA, USA) to remove any contaminating genomic DNA and enriched for coding and non-coding transcriptomes using Ribo-Zero ribosomal RNA removal kit (Illumina, San Diego, CA, USA). The quantity and quality of enriched total RNA was ascertained, respectively, on a Multiskan^TM^ Go Spectrophotometer (Thermo Fisher Scientific) and Bioanalyzer (Agilent Technologies, Santa Clara, CA, USA), and only RNA samples with an RNA integrity number (RIN) of >9 were included in our experiments. The enriched RNA was subjected to reverse transcription, followed by preparation of barcoded and strand-specific complementary DNA (cDNA) libraries using TruSeq RNA Sample Prep Kit (Illumina), as described earlier [30]. The cDNA libraries were then processed for sequencing as 50-base-long paired-end reads on an Illumina HiSeq 1500 at the Next Generation Sequencing core facility of the UTMB. Three independent biological replicates for each experimental condition (i.e., mock- and *R. conorii*-infected) were subjected to library preparation and RNA-seq in tandem. The RNA-seq data is available in the GenBank (Accession number GSE121808).

### 2.4. Mapping of Deep Sequencing Data and Ingenuity Pathway Analysis

The CLC Genomics Workbench v11.0.1 (Qiagen, Redwood City, CA, USA) was used for bioinformatic analysis of the RNA-seq data. Sequencing data was initially trimmed using the “Trim Reads” module. The reads containing nucleotides below the quality threshold of 0.05 (using the modified Richard Mott algorithm) and those with two or more unknown nucleotides or sequencing adapters were trimmed out. The total number of reads from individual samples used in the analysis ranged from 40 to 70 million. Filtered sequencing reads were next processed using the “RNA-seq Analysis” module. The reads were aligned against the *Mus musculus* (GRCm38) reference genome with an annotated gene track. The minimum matching length and similarity fraction was 90%. Principal component analysis (PCA) was performed to cluster by the similarity of individual sample gene expression profiles. Differential gene expression test was conducted using multi-factorial statistics based on a negative binomial generalized linear model (GLM). Ingenuity pathway analysis (IPA) was performed on gene expression profiles generated by CLC Genomics RNA-seq Analysis. Features with a mean expression value of less than 10, a *p*-value above 0.05, and less than a 1.5-fold change value were excluded from the analysis. A total of 1937 qualifying features based on these described analysis criteria were subjected to IPA.

### 2.5. Real-Time Quantitative Polymerase Chain Reaction (RT-qPCR)

Total RNA from the mock- and *R. conorii*-infected mouse lungs (*n* ≥ 4) was prepared using the Tri-reagent protocol described above. The total RNA was incubated with DNaseI (New England Biolabs, Ispwich, MA, USA) to remove genomic DNA contamination, and it was precipitated using approximately 1/10th volume of 3 M sodium acetate (pH 5.2) and glycogen (10 µg; Thermo Scientific) following standard laboratory protocols [28]. The purified total RNA was quantified on a Multiscan^TM^ Go spectrophotometer (Thermo Scientific) and the samples yielding a 260/280 and 260/230 ratio ranging between 1.8 and 2.2 were used for RT-qPCR. Complementary DNA was synthesized using the High Capacity cDNA synthesis kit (Applied Biosystems, Foster City, CA, USA) following manufacturer instructions. Briefly, about 1 µg of total RNA was reverse-transcribed in a 20 µl reaction volume containing random primer at a final concentration of 5 µM, reverse transcriptase (50 units), and dNTP mix (4 mM) with the cycling conditions set to 25 °C for 10 min, 37 °C for 60 min, and 85 °C for 5 min. Quantification of target genes from the cDNA template was performed using PowerUp^TM^ SYBR^®^ Green master mix (Applied Biosystems) on a StepOne Plus real-time PCR system (Applied Biosystems) with each primer at a final concentration of 0.4 µM. Cycling conditions for the PCR segment of SYBR^®^ Green assay were 95 °C for 10 min, 40 cycles of 95 °C for 15 s and 60 °C for 1 min, followed 95 °C for 15 s, 60 °C for 1 min, and a melt curve. For each target gene, the samples as biological replicates (*n* ≥4) were assayed in technical triplicates to account for any intra-sample variations. Also, the biological replicates for each target gene and 18S rRNA as the endogenous control were run on the same PCR plate to negate any inter-run variations. The absence of genomic DNA contamination in the RNA samples was verified via a no-RT control on each plate. The cycle number for the detection threshold (*C_T_*) values of the target genes were normalized to the *C_T_* of 18S rRNA (endogenous control) using the ∆∆*C_T_* method [31], and the fold-change values in *R. conorii*-infected over mock-infected samples were calculated as Log_2_ fold (Log_2_ 3.32 = 10 fold change). All primer sequences used in this study are listed in Table S1.

### 2.6. Statistical Analysis

All RT-qPCR experiments were performed on a minimum of four independent biological replicates with three technical replicates. The expression (Log_2_ fold) of target genes in *R. conorii*-infected samples relative to the control (mock-infected) was determined as the mean ± standard error of the mean (SEM). Statistical analysis was performed using GraphPad Prism v4.0 (GraphPad, San Diego, CA, USA), with significance determined by Mann–Whitney U test. A *p*-value of ≤0.05 was considered to represent a significant change.

## 3. Results

### 3.1. Transcriptome Sequencing, Assembly, and Analysis of Mouse Lungs During In Vivo R. conorii Infection

Pathogenic *Rickettsia* species are capable of infecting different cell types, including macrophages, hepatocytes, fibroblasts, and vascular endothelial cells lining capillaries and arterioles, and target all vital organ systems including the brain, heart, kidneys, lungs, and testes of the mammalian hosts. In humans and experimental laboratory models of infection, however, infection of microvascular endothelial layers of blood vessels, resulting in vascular inflammation and dysfunction and manifesting as maculopapular rash, acute respiratory distress syndrome, myocarditis, and meningoencephalitis, represent the prominent, characteristic features of pathogenesis. In the present study, we determined the changes in the global transcriptome of lungs as one of the primary target organs in an established murine model of *R. conorii* infection. We performed deep, paired-end sequencing on RNA samples from the lungs of control (mock-infected) and *R. conorii*-infected C3H/HeN mice. Sequencing of cDNA libraries resulted in an average of 50.8 and 53.4 million reads from the control (mock-infected) and *R. conorii*-infected lung tissue, respectively, of which approximately 97% of the reads mapped with 100% identity to *Mus musculus* (mm9) genome (Table S2). To analyze changes in the host’s coding transcriptome as a result of *R. conorii* infection, we mapped the read libraries to annotated coding genes in the NCBI reference sequence database (Ref-Seq) to find that about 30.0% and 32.5% of total reads, respectively, from the RNAs derived from the mock- and *R. conorii*-infected mouse lungs corresponded to protein coding genes. Overall, our analysis revealed altered expression in a total of 1332 genes, including 659 up-regulated and 132 down-regulated transcripts based on a cut-off threshold of ±2 log-fold (*p* ≤ 0.05). Most notable among these were the genes encoding for ubiquitin D, aconitate decarboxylase, antimicrobial peptides, cytokines and chemokines, and guanylate binding proteins displaying a pattern of high levels of expression (up-regulation). Genes (*Hbb-bt*, *Hba1/Hba2*, and *Alas2*) involved in hemoglobin biosynthesis and heme homeostasis, on the other hand, were significantly down-regulated in response to infection (Table 1 and Table S3).

### 3.2. Validation of RNA-Sequencing Data by Quantitative Real-Time PCR

**Figure 1:**
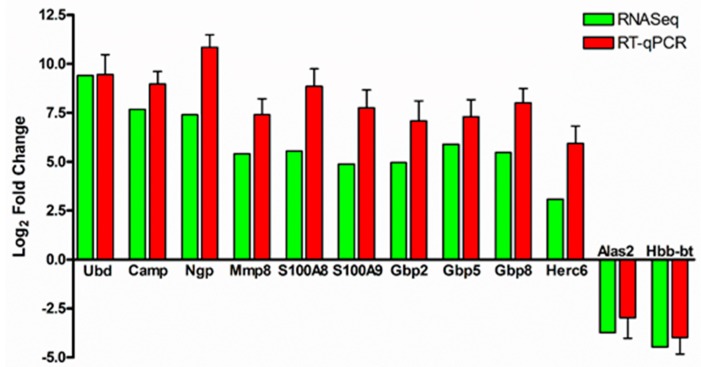
Validation of RNA-seq data by real time quantitative PCR (RT-qPCR). Total RNA from the lungs of mock-infected controls and *Rickettsia conorii*-infected mice was extracted by Tri-reagent, DNaseI treated, and reverse transcribed. Quantitative RT-PCR was performed using gene-specific primers and 18S rRNA as the endogenous control. Gene expression observed in RNA-seq data sets are presented as mean, and RT-qPCR as the mean ± standard error of the mean (SEM). The fold change observed in RT-qPCR is in accordance with that observed in RNA-seq data.

We next performed quantitative RT-PCR to validate changes in the expression of 12 genes determined to be differentially expressed during *R. conorii* infection in our RNA-seq datasets. To this end, total RNA from the lungs of mock- and *R. conorii*-infected mice was subjected to DNaseI treatment to digest any contaminating genomic DNA, and it was reverse transcribed following previously established protocols and procedures [28]. Gene expression was quantified using SYBR^®^ Green real-time PCR master mix containing primers specific to the genes of interest and 18S rRNA as an endogenous housekeeping control. All ten up-regulated genes, namely *Ubd*, *Camp*, *Ngp*, *Mmp8*, *S100A8*, *S100A9*, *Gbp2*, *Gbp5*, *Gbp8,* and *Herc6*, and both down-regulated genes (*Alas2* and *Hbb-bt*) exhibited a similar pattern of expression in RT-qPCR assays, confirming our initial findings from the RNA-seq analysis (Figure 1).

### 3.3. Functional Annotation and Classification of Transcriptome Data

**Figure 2:**
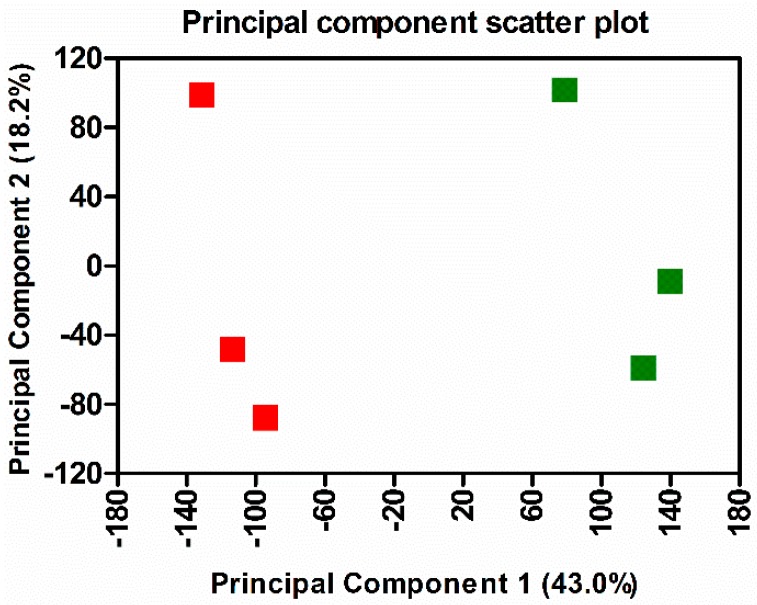
Principal component analysis of gene expression in the lungs of mock-infected controls and *R. conorii*-infected C3H/HeN mice at three days post-infection. Green: mock-infected control, Red: *R. conorii*-infected.

**Figure 3:**
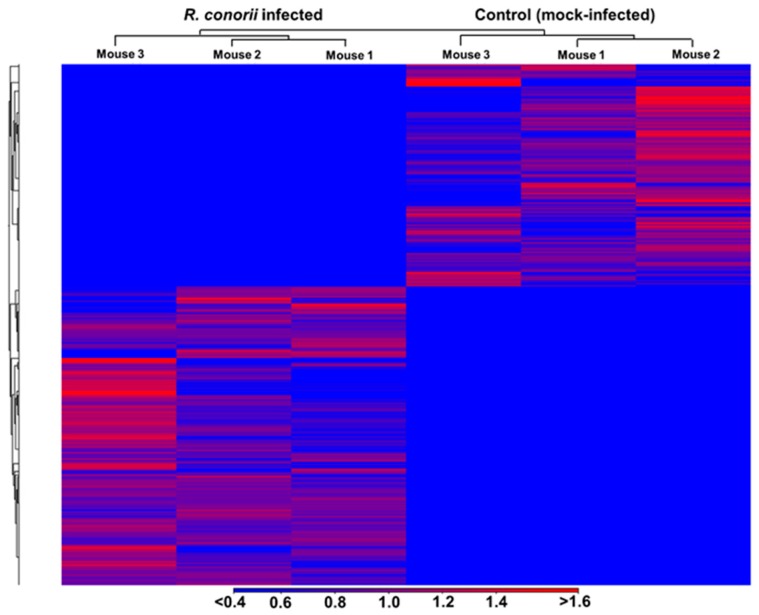
Hierarchical clustering of differentially expressed genes in the lungs of control and *R. conorii*-infected mice. Red color in the cluster indicates up-regulation and blue color in the cluster indicates down-regulation. The fold expression values presented below are in terms of log_2_.

Hierarchical clustering and principal component analysis of differentially expressed genes revealed clear demarcation of hierarchical clusters and groups with a high degree of variance between the mock- and *R. conorii* infected samples (Figure 2 and Figure 3). We next employed IPA (Qiagen, Redwood City, CA, USA) to perform functional classification of differentially expressed genes to delineate the activation or inhibition of upstream regulators and to enrich the pathways subjected to their regulatory effects. We were able to identify differential regulation of 292 regulators, of which 94 were activated and 37 were inhibited due to *R. conorii* infection based on ≥±2 activation *z*-score (*p* ≤ 0.05) (Table 2 and Table S4). Functional annotation analysis revealed significant up-regulation of 196 functional categories (≥2 activation z-score, *p* ≤ 0.05) involved in the biological processes of cell movement, chemotaxis, homing of blood cells, phagocytes and leukocytes, inflammatory responses, cell-to-cell signaling and interactions, recruitment of granulocytes and neutrophils, and adhesion of immune cells. Only eight functional annotation categories, including growth of bacteria and protein translation, exhibited a state of decreased activation (≤−2 activation *z*-score, *p* ≤ 0.05) (Table S5).

### 3.4. Signatures of Inflammation and Tissue Damage

Functional classification of genes and pathways by IPA demonstrated that several cytokines, enzymes (including kinases), growth factors, G-protein-coupled receptors, and interferon-induced genes were differentially expressed during *R. conorii* infection. Amongst cytokines, the genes for *Cxcl9*, *Cxcl10*, *Cxcl13*, *Il-1β*, *Il-6*, *Tnf*, *Tnfsf10*, *IFN-γ, Ccl2*, *Ccl4*, *Ccl7*, *Ccl17*, and *Mif* were highly up-regulated (ranging from 2- to 405-fold, *p* ≤ 0.05), whereas *Cxcl12* and *Cxcl14* were down-regulated during in vivo infection (Table 1 and Table S3). In addition, several other interferon-stimulated genes, including *Isg15*, *Isg20*, *Ifi35*, *Ifi44*, *Ifi47* and *Ifi202b*, *Ifit1B*, *Ifit1*, *Ifit2* and *Ifit3*, and *Igtp* were also highly expressed at greater than 2-fold over basal expression in mock-infected controls. Interestingly, Ido-1, which is known to restrict bacterial growth and facilitate immune tolerance by modulating T-cell functions, displayed 332-fold higher expression in mice infected with *R. conorii* when compared to the corresponding controls. Furthermore, several genes involved in apoptosis, namely *Bak1*, *Bcl2A1*, *Birc2* and *Birc3*, *Casp1*, *Casp4*, *Casp7* and *Casp8*, *Ecscr*, *Fas*, *Nfkb*, *Tnf*, and *Tnfaip3* were up-regulated, suggesting activation of apoptotic machinery and tissue damage (Table S3). The steady-state expression of *Tlr2*, *Tlr4*, and *Tlr13* was determined to be increased by 3.2-, 2.0-, and 4.5-fold over their respective basal levels during *R. conorii* infection in vivo. Interestingly, expression of several calcium binding proteins, including *S100A4*, *S100A8*, *S100A9,* and *S100A10*, was significantly increased (4.6- to 46.9-fold, *p* ≤ 0.05) during *R. conorii* infection. In addition, the gene coding for *Calhm6*, a subunit of the voltage-gated ion channel involved in calcium homeostasis, was also abundantly expressed (52-fold higher than the controls, *p* ≤ 0.05) during rickettsial infection. Finally, we also observed a robust elevation in the expression of guanylate binding proteins (*Gbp*) as illustrated by significantly higher levels (14- to 127-fold, *p* ≤ 0.05) of different Gbps (including *Gbp2*, *Gbp3*, *Gbp4*, *Gbp5*, *Gbp6*, *Gbp7*, and *Gbp8*) during in vivo rickettsial infection (Table 1, Figure 1, and Table S3). In line with these findings, the ‘growth of bacteria’ turned out to be one of the most inhibited functional annotation categories in this study (Table S5).

### 3.5. Antimicrobial Peptides

Antimicrobial peptides (AMPs), also known as host defense peptides, are primarily found within the granules of neutrophils. They disrupt physical integrity of the microbial cellular membrane upon activation by proteolytic cleavage, thereby resulting in pathogen clearance. Strikingly, our analysis revealed significantly higher levels of expression of two AMPs, namely cathelicidin antimicrobial peptide (*Camp*) and neutrophilic granule protein (*Ngp*), as evidenced by increases of 203- and 167-fold (*p* ≤ 0.05), respectively, in the lungs of *R. conorii*-infected mice, when compared to simultaneously processed mock-infected controls (Table 1 and Figure 1). However, we did not observe noticeable changes in the expression pattern of defensin peptides, indicating only selective expression of AMPs during rickettsial infection.

### 3.6. Activation or Inhibition of Upstream Signaling Regulators

We next utilized IPA to determine the activation or inhibition status of the upstream regulators in host signaling pathways. This resulted in the identification of a total of 94 activated and 37 inhibited upstream regulators based on the *z*-score of ±2 and *p* ≤ 0.05 (Table S4). The representative activated genes included *Myd88*, *Stat1*, *Mavs*, *Irf3*, *Irf5*, *Irf7*, *Tlr4*, *Ifn-γ*, *Nfatc2*, and *Ticam1*, while *Il10ra*, *Bcl6*, *Tgfbr1*, *Stat6*, *Socs1*, *Irgm1*, and *Ptger4* were amongst the list of top 20 inhibited upstream regulators based on the activation *z*-score of ±2 and significant cut-off of *p* ≤ 0.05 (Table 2). Additionally, a number of other upstream regulators such as *Tlr3*, *Tlr7*, *Ifnb*, *Ifnar1*, *Nod2*, *Parp1*, *Mapk*, *Klrk1* and *Klrk3*, and *Tbk1* were also found to be activated during in vivo rickettsial infection.

### 3.7. Canonical Pathways

We next clustered genes exhibiting significant alterations in their expression during *R. conorii* infection by ingenuity pathway enrichment analysis into biological canonical pathways. Five robustly up-regulated pathways with the highest number of differentially expressed genes were categorized into eukaryotic initiation factor 2 (EIF2) signaling, oxidative phosphorylation, mitochondrial dysfunction, antigen presentation pathway, and protein ubiquitination pathway (Table 3). A total of 23 genes (82%) representative of the antigen presentation pathway and 72 of 245 genes in the protein ubiquitination pathway were differentially expressed during rickettsial infection. Notably, ubiquitin D was determined to be the top most significantly expressed gene, exhibiting about 680-fold (*p* ≤ 0.05) up-regulation in lungs of *R. conorii*-infected hosts. A number of other genes involved in protein ubiquitination pathways, for example *Ube2l6*, *Uba7*, *Usp18*, *Nedd8*, *Cdc34*, and *Herc6,* were also dramatically up-regulated, implicating a potentially important role for this pathway in host response to rickettsial infection (Table 1, Figure 1, and Table S3). The EIF2 signaling pathway and oxidative phosphorylation also had higher *p*-values of 1.54 × 10^−42^ and 1.29 × 10^−21^, respectively, when compared to other three pathways, and approximately 50% of the genes were differentially expressed in both of these pathways.

## 4. Discussion

*Rickettsia* species belonging to the spotted fever group are prevalent worldwide and are increasingly recognized as the causative agents of emerging infectious diseases in humans [1,32,33]. Although pathogenic rickettsiae are capable of infecting several different host cell types, including hepatocytes, macrophages, and vascular smooth muscle cells, established primary targets of infection in the mammalian hosts are vascular endothelial cells lining the vessels of all major tissues and organ systems. Consequently, in patients with RMSF (*R. rickettsii*) and MSF (*R. conorii*)*,* damage to the vascular endothelium of vital organ systems, such as the brain, heart, lungs, kidneys, and gastrointestinal tract, leads to moderate to severe systemic manifestations, characteristically referred to as rickettsial vasculitis [34]. A number of previous studies, including those from our laboratory, have heretofore focused on profiling gene expression patterns and host defense mechanisms in cultured human endothelial cells or macrophages infected in vitro with different *Rickettsia* species. In a recent study, Riley et al. have also evaluated changes in the pathogen transcriptome during *R. rickettsii* infection of an experimental murine host, but analysis of qualitative and quantitative differences in the transcriptome of target host organs has not yet been addressed [35]. We present here, for the first time, a comprehensive assessment of gene expression patterns in the lungs of susceptible mammalian hosts (C3H/HeN mice) during *R. conorii* infection. This is an established small animal model capable of recapitulating the pathogenesis of spotted fever rickettsioses in humans. Our rationale for the organ of choice to begin these studies was based on the significant pathogen burden in the lungs of *R. conorii*-infected C3H/HeN mice [6,18] and published evidence implicating considerable pulmonary involvement in the pathophysiology of human rickettsioses [36]. As such, one of the major objectives of this study was to discover unrealized genes or neglected biological pathways, which might play important roles in the determination of host responses and rickettsial pathogenesis. In that regard, presented findings identify unique sets of genes and link them together as potential determinants of important regulatory pathways, further analysis of which will expand our understanding of the mechanisms underlying host immune response to rickettsial infections.

Previous in vitro, in vivo, and clinical studies have documented that *R. conorii* infection induces a variety of cytokines and chemokines (e.g., IFN-γ, CXCL9 (MIG), CXCL10 (IP-10), and CCL2 (MCP-1)) involved in the governance of immune responses in both humans and animals [1,5]. As expected, the expression of these genes was determined to be significantly up-regulated in the present study. Interestingly, however, the genes coding for ubiquitin D, cathelicidin antimicrobial peptide (CAMP), neutrophil granule protein (NGP), lymphocyte antigen 6 complex, locus A (Ly6a), calcium binding protein (S100A8), calcium and zinc binding protein (S100A9), guanylate binding proteins, and indoleamine 2,3-dioxygenase were highly up-regulated in response to infection. Ubiquitin D, a highly conserved 165-residue (18kDa) protein found in the cytoplasm and nucleus of eukaryotic cells, is involved in the regulation of cell cycle and proliferation, immunoproteasome formation, and antigen presentation, and contributes to inflammatory reactions by mediating NF-κB activation. During human immunodeficiency viruses (HIV)-induced nephropathy, overexpressed ubiquitin D reportedly induces apoptosis by interacting with HIV Vpr protein [37,38]. CAMP (also known as LL-37) displays both antimicrobial activities, such as direct killing of bacteria via osmotic lysis and immunoregulatory activities via induction of autophagy, regulation of chemokine production and chemokine receptor expression, modulation of cytokine secretion, and chemotactic effects on immune cells [39]. Neutrophils and alveolar macrophages infected in vitro with *Mycobacterium tuberculosis* have been shown to produce CAMP [40]. Neutrophils are the prime orchestrators of lung inflammation by virtue of their actions linking innate and adaptive immunity, and lungs are known to be the reservoir of neutrophils under steady-state conditions. Because antimicrobial activities of neutrophils are predominantly attributed to the release of granule proteins such as myeloperoxidase, induced expression of neutrophil granule protein (NGP) in *R. conorii*-infected mouse lungs would suggest their involvement in pathogen clearance and host defense. It is also possible, however, that extensive recruitment and activation of neutrophils may exacerbate tissue damage, as has been shown to occur in murine models of *R. typhi* and *Orientia tsutsugamushi* infections [41,42]. Ly6a [Stem cell antigen-1 (Sca-1)], a glycosylphosphatidylinositol-anchored protein expressed on many cell types including hematopoietic stem cells, early lymphoid-specific progenitors, and T cells, plays a critical role in regulating cellular responses to foreign antigens. Because both CD4 and CD8 T cells contribute to cell-mediated immunity during rickettsial infections [43,44,45], high levels of pulmonary Ly6a expression likely reflect increases of infiltrating T cells recruited to host lungs during *R. conorii* infection. Taken together, our results clearly suggest the involvement of antimicrobial peptides as part of the host innate defense and highlight the need for further exploration of this previously unappreciated aspect in the control of rickettsial infections.

S100A8 and S100A8 belong to the S100 superfamily of proteins representing the largest subgroup of Ca^2+^-binding proteins of the EF-hand type. They are also termed calgranulins because of their Ca^2+^ binding properties and high expression levels in activated granulocytes. Both S100A8 and S100A8 are expressed in cells and tissues and perform a number of intra- and extracellular functions, which include cytokine- and chemokine-like activities via activation of the receptor-associated signaling cascades. S100A8 and S100A9 are cytoplasmic, phagocyte-specific, and death-associated molecular patterns capable of forming stable heterodimers called calprotectin because of its protective, anti-microbial effects. Since pathogenic versus protective effects of S100A8 and S100A9 occur in a context-specific manner and tend to vary depending on the state of inflammatory milieu, further detailed studies to identify their source of origin during rickettsial infections and to determine their roles in disease pathogenesis or host defense mechanisms are necessary.

Guanylate binding proteins are a family of dynamin-related, interferon-inducible GTPases that have central roles in cell-autonomous immunity, and they are induced by LPS and other stimuli such as IFN-γ. GBPs coordinate a wide array of innate immune functions against intracellular pathogens and are known to exert antimicrobial mechanisms against pathogens with diverse intracellular lifestyles, including viruses, bacteria, and protozoa. Recent studies have suggested that bacterial pathogens, for example *Shigella flexneri*, utilize specialized secretion system effectors to degrade host GBPs to interfere with the antibacterial defense [46,47]. During infection with the cytosolic bacterium *Francisella tularensis*, GBPs acting downstream of transcription factor IRF1 involved in type I interferon signaling are involved in the activation of AIM2 inflammsome, release of bacterial DNA as a pathogen-associated recognition pattern, and antibacterial activities [48,49]. In a previous in vitro study based on the infection of human endothelial cells with *R. conorii*, we have identified increased expression of GBP1 independent of IFN-β expression [21], and the activation of inflammasome by the host as an anti-rickettsial strategy has also been demonstrated [50]. Our current analysis suggests induced expression of another subset of GBPs, including GBP2 through GBP8, in the lungs of infected hosts. Considering that immune GTPases serve in the stimulation of autophagic, membranolytic, oxidative, and inflammasome-mediated antimicrobial functions within the cytosol, systematic in-depth analysis aimed at the identification of GBP-interacting proteins will likely reveal additional anti-rickettsial defense programs regulated by GBPs, which could then be exploited for the design and development of new therapeutics. Also, whether microbial molecules such as lipopolysaccharide (LPS) or host–response pathways promote GBP activation and function is another unanswered question surrounding these critical mediators of host resistance and inflammation. Finally, our finding of increased expression of indoleamine-2,3-deoxygenase, an enzyme involved in tryptophan degradation, is in agreement with published observations of increased intralesional expression in skin biopsies from patients with MSF and its role in preventing rickettsial growth and replication in macrophages [17].

Kyoto Encyclopedia of Genes and Genomes (KEGG) pathway enrichment analysis of differentially expressed genes to achieve a broader understanding of the host lungs’ immune responses following *R. conorii* infection revealed the activation of TNF, NF-κB, JAK-STAT, MAP Kinase, toll-like receptor, and NOD-like receptor signaling, as well pathways governing the cytokine/chemokine activities and apoptosis. As would be expected, a large number of differentially expressed genes were found to be associated with the antigen presentation pathway, an important component of the pathogen recognition and host defense against infection. Interestingly, protein ubiquitination, oxidative phosphorylation, and EIF2 signaling pathways were also enriched in our datasets, suggesting the potential contribution to anti-rickettsial strategies in the host. Although activation of EIF2 signaling has been implicated in opposing invasion of host cells by intracellular bacterial pathogens of *Listeria* and *Chlamydia* species [51], its importance in rickettsial interactions with the host remains completely unknown. Similarly, protein ubiquitination has been proven to be a critical signal for the regulation of a number of cellular processes such as cell cycle progression, DNA repair, immune response, and vesicular trafficking. Therefore, it is not surprising that a plethora of pathogens utilize specialized strategies to interact with and exploit the host ubiquitin system to their advantage. An important consideration in this regard is that the host ubiquitinome is one of the major determinants of actin cytoskeleton, NF-κB response, and autophagy pathways, all of which have been shown to contribute to host–pathogen interactions during rickettsial infections [52,53,54,55,56]. Further investigation of an interplay with host mechanisms regulated by ubiquitination will add a new dimension to the biology of rickettsial infections. An enhanced understanding of this aspect could be vital for defining potentially novel therapeutic intervention strategies against these pathogens.

## Figures and Tables

**Table 1:** List of the top 20 up- and down-regulated molecules in mouse lung tissue during in vivo *R. conorii* infection.

Symbol	Gene Name	*p*-Value	FDR (*q*-Value)	FC	Log_2_ FC
**Up-regulated molecules**					
*Ubd*	Ubiquitin D	0	0	679.8	9.4
*Acod1*	Aconitate decarboxylase 1	0	0	433.9	8.8
*Cxcl9*	Chemokine (C-X-C motif) ligand 9	0	0	405.4	8.7
*Ido1*	Indoleamine 2,3-dioxygenase 1	0	0	332.3	8.4
*Cxcl10*	C-X-C motif chemokine ligand 10	0	0	331.6	8.4
*Ly6a*	Lymphocyte antigen 6 complex, locus A	0	0	223.2	7.8
*Camp*	Cathelicidin antimicrobial peptide	0	0	204	7.7
*Ngp*	Neutrophilic granule protein	0	0	168	7.4
*Gzmk*	Granzyme K	0	0	135.7	7.1
*Gbp6*	Guanylate binding protein family member 6	0	0	127.8	7
*Ifn-γ*	Interferon γ	0	0	119.8	6.9
*Serpina3g*	Serine (or cysteine) peptidase inhibitor, clade A, member 3G	0	0	117.5	6.9
*Gzmb*	Granzyme B	0	0	103.9	6.7
*Gzma*	Granzyme A	0	0	90.4	6.5
*Klrg1*	Killer cell lectin like receptor G1	0	0	87.7	6.5
*Ccl7*	Chemokine (C-C motif) ligand 7	0	0	83.9	6.4
*Ccl2*	Chemokine (C-C motif) ligand 2	0	0	74.8	6.2
*Tgtp1*/*Tgtp2*	T cell specific GTPase 1	0	0	73.4	6.2
*Gbp5*	Guanylate binding protein 5	0	0	59.1	5.9
*Xcl1*	X-C motif chemokine ligand 1	0	0	52.6	5.7
*Calhm6*	Calcium homeostasis modulator family member 6	0	0	52.2	5.7
**Down-regulated molecules**					
*Hbb-bt*	Hemoglobin subunit β	0	0	−25.2	−4.7
*Hba1*/*Hba2*	Hemoglobin subunit α 2	0	0	−22	−4.5
*Alas2*	5’-aminolevulinate synthase 2	0	0	−13.3	−3.7
*Cyp26b1*	Cytochrome P450 family 26 subfamily B member 1	3 × 10^−7^	0.000005	−10.2	−3.3
*Alb*	Albumin	0.000546	0.00461	−9.2	−3.2
*Hey1*	Hes related family bHLH transcription factor with YRPW motif 1	0	0	−6.9	−2.8
*Aplnr*	Apelin receptor	0	0	−6.6	−2.7
*Nr1d1*	Nuclear receptor subfamily 1 group D member 1	0	0	−5.9	−2.6
*Ptprb*	Protein tyrosine phosphatase, receptor type B	0	0	−5.8	−2.5
*Dll4*	Delta like canonical Notch ligand 4	0	0	−5.7	−2.5
*Efnb2*	Ephrin B2	0	0	−5.1	−2.3
*Ltbp4*	Latent transforming growth factor β binding protein 4	0	0	−5	−2.3
*Hmcn1*	Hemicentin 1	0	0	−4.9	−2.3
*Angptl2*	Angiopoietin like 2	0	0	−4.7	−2.2
*Ednrb*	Endothelin receptor type B	0	0	−4.1	−2
*Spock2*	SPARC/osteonectin, cwcv and kazal like domains proteoglycan 2	0	0	−4.1	−2
*Sh3pxd2a*	SH3 and PX domains 2A	0	0	−3.7	−1.9
*Ndst1*	N-deacetylase and N-sulfotransferase 1	0	0	−3.7	−1.9
*Igfbp5*	Insulin like growth factor binding protein 5	6.68 × 10^−7^	1.04 × 10^−5^	−3.5	−1.8
*Pdgfrb*	Platelet derived growth factor receptor β	0	0	−3.5	−1.8

FDR: False Discovery Rate; FC: Fold Change; Log_2_ FC: Log_2_ Fold Change.

**Table 2:** List of the top 20 upstream regulators activated or inhibited during in vivo *R. conorii* infection.

Regulator	Molecule Type	*p*-Value	Activation *z*-Score
**Activated upstream regulators**			
*Irf3*	Transcription regulator	2.21 × 10^−24^	6.8
*Ifn-γ*	Cytokine	1.82 × 10^−^^27^	6.5
*Ticam1*	Other	4.13 × 10^−^^22^	6.3
*Irf7*	Transcription regulator	1.62 × 10^−24^	5.8
*Stat1*	Transcription regulator	1.62 × 10^−17^	5.7
*Myd88*	Other	8.02 × 10^−20^	5.5
*Cd38*	Enzyme	1.17 × 10^−8^	5.5
*Samsn1*	Other	5.86 × 10^−12^	5.4
*Il5*	Cytokine	3.07 × 10^−9^	5.2
*Rb1*	Transcription regulator	8.56 × 10^−6^	4.9
*Dock8*	Other	5.24 × 10^−11^	4.9
*Sash1*	Other	1.03 × 10^−9^	4.8
*Tlr4*	Transmembrane receptor	4.04 × 10^−16^	4.7
*Nfatc2*	Transcription regulator	1.88 × 10^−8^	4.6
*Tnf*	Cytokine	3.54 × 10^−13^	4.6
*Chuk*	Kinase	9.36 × 10^−14^	4.5
*Mavs*	Other	1.04 × 10^−14^	4.3
*Arhgap21*	Other	7.33 × 10^−7^	4.1
*Ikbkb*	Kinase	8.43 × 10^−17^	4.1
*Irf5*	Transcription regulator	2.48 × 10^−9^	4
**Inhibited upstream regulators**			
*Il10ra*	Transmembrane receptor	1.85 × 10^−19^	−7.4
*Ptger4*	G-protein coupled receptor	9.94 × 10^−16^	−6
*Kdm5a*	Transcription regulator	4.61 × 10^−7^	−5.2
*Socs1*	Other	1.19 × 10^−15^	−4.6
*Irgm1*	Other	2.56 × 10^−10^	−4
*Bcl6*	Transcription regulator	7.76 × 10^−5^	−3.6
*Srf*	Transcription regulator	0.000721	−3.4
*Nr3c1*	Ligand-dependent nuclear receptor	7.27 × 10^−8^	−3.4
*Gfi1*	Transcription regulator	0.000536	−3.3
*Ncstn*	Peptidase	2.48 × 10^−9^	−3.3
*Apoe*	Transporter	5.75 × 10^−6^	−2.9
*Por*	Enzyme	5.08 × 10^−5^	−2.9
*Dusp1*	Phosphatase	0.013	−2.7
*C5*	Cytokine	0.0151	−2.6
*Tgfbr1*	Kinase	0.0333	−2.6
*Dicer1*	Enzyme	0.00974	−2.6
*Stat6*	Transcription regulator	5.24 × 10^−14^	−2.5
*Cyb5r4*	Enzyme	0.000106	−2.4
*Abcg1*	Transporter	0.00048	−2.4
*Irf9*	Transcription regulator	2.32 × 10^−8^	−2.3

**Table 3:** List of five canonical pathways with the highest number of differentially expressed genes during in vivo *R. conorii* infection.

Canonical Pathway Name	*p*-Value	Differentially Expressed Genes/Total Number of Genes in the Pathway
EIF2 Signaling	1.54 × 10^−42^	100/205 (48.8%)
Oxidative Phosphorylation	1.29 × 10^−21^	48/96 (50.0%)
Mitochondrial Dysfunction	3.29 × 10^−19^	59/153 (38.6%)
Antigen Presentation Pathway	3.90 × 10^−18^	23/28 (82.1%)
Protein Ubiquitination Pathway	1.33 × 10^−15^	72/245 (29.4%)

## Supplementary Materials

The following are available online at https://www.mdpi.com/2073-4425/10/3/204/s1, Table S1: Listed of primers used in this study, Table S2: Summary of RNA Sequencing read counts and mapping statistics, Table S3: List of genes differentially expressed during *R. conorii* infection in vivo, Table S4: List of upstream regulators differentially regulated during the infection of *R. conorii* in vivo, Table S5: List of functional annotations exhibiting increased or decreased activation state in mouse lung tissue during *R. conorii* infection in vivo.
